# Profiling the Metabolism of Astragaloside IV by Ultra Performance Liquid Chromatography Coupled with Quadrupole/Time-of-Flight Mass Spectrometry

**DOI:** 10.3390/molecules191118881

**Published:** 2014-11-17

**Authors:** Xu-Dong Cheng, Ming-Gang Wei

**Affiliations:** 1 College of Pharmacy, Nanjing University of Chinese Medicine, No. 218 Xianlin Avenue, Nanjing 210046, China; E-Mail: chengxd2013@gmail.com; 2 The First Affiliated Hospital of Soochow University, No. 188 Shizi Street, Suzhou 215006, China

**Keywords:** astagaloside IV, metabolite profiling, UPLC-Q-TOF-MS/MS

## Abstract

Astragaloside IV is a compound isolated from the Traditional Chinese Medicine *Astragalus membranaceus*, that has been reported to have bioactivities against cardiovascular disease and kidney disease. There is limited information on the metabolism of astragaloside IV, which impedes comprehension of its biological actions and pharmacology. In the present study, an ultra-performance liquid chromatography coupled with quadrupole/time-of-flight mass spectrometry (UPLC-Q-TOF-MS/MS)-based approach was developed to profile the metabolites of astragaloside IV in rat plasma, bile, urine and feces samples. Twenty-two major metabolites were detected. The major components found in plasma, bile, urine and feces included the parent chemical and phases I and II metabolites. The major metabolic reactions of astragaloside IV were hydrolysis, glucuronidation, sulfation and dehydrogenation. These results will help to improve understanding the metabolism and reveal the biotransformation profiling of astragaloside IV *in vivo*. The metabolic information obtained from our study will guide studies into the pharmacological activity and clinical safety of astragaloside IV.

## 1. Introduction

The metabolism of drugs in organisms is an important biotransformation process. The study of the metabolite profile of a candidate compound plays an important part in demonstrating the basic information about a biological process as well as the pharmacology and toxicology of a drug [1]. Detection and characterization of metabolites using appropriate methods are important for the assessment of the hidden risks of a drug [2]. Preclinical metabolism studies are used to assess the absorption, distribution, metabolism, excretion and toxicity profiles of new candidate drugs to ascertain if they should be developed further [3]. Profiling of the metabolites of Traditional Chinese Medicines (TCMs) is also being studied, which is important for their development [4].

Astragaloside IV is one of the major active saponins isolated from the roots of the TCM *Radix astragali* [5]. Studies have shown that astragaloside IV can protect against ischemic brain injury [6] and has certain other interesting effects (anti-inflammatory [7], anti-renal injury [8], anti-scarring [9], immunoregulatory [10]). Studies focusing on the pharmacokinetics and bioavailability of astragaloside IV suggest that astragaloside IV has low bioavailability after oral administration [11,12,13]. The mechanism of absorption of astragaloside IV has been studied [14]. The metabolic profile of astragaloside IV appears to be ambiguous. The metabolism of a complex of herbs containing *Radix astragli* in rats has been studied. It has been reported previously that metabolites of saponins in plasma, urine and bile samples of rats after administration of Danggui Buxue Tang (DBT) via hydrolysis, glucuronic acid and dehydrogenation of metabolic pathways [15]. However, trace levels of astragaloside IV and no metabolites were detected in rat urine samples after administration of Buyang Huanwu decoction (BYHWD), a TCM with eight herbs containing mainly *Radix astragli* [16]. In TCMs, the metabolism of astragaloside IV could be affected by other compounds and may not be able to be detected at low concentrations. To understand the mechanism of the transformation of astragaloside IV after administration, metabolite studies must be carried out.

In the present study, the metabolite profile of astragaloside IV was investigated with ultra-performance liquid chromatography coupled with electrospray ionization quadrupole time-of-flight tandem mass spectrometry (UPLC-Q-TOF-MS/MS) in rats using plasma, bile, urine, and feces samples. The phase I and phase II metabolites were detected and identified using the MetaboLynx software (Waters, Milford, MA, USA). Twenty two metabolites were detected and most of them characterized for the first time. The metabolic pathways were revealed for better understanding of the pharmacological process of astragaloside IV.

## 2. Results and Discussion

### 2.1. Chromatographic and MS Characterization of Astragaloside IV

**Figure 1:**
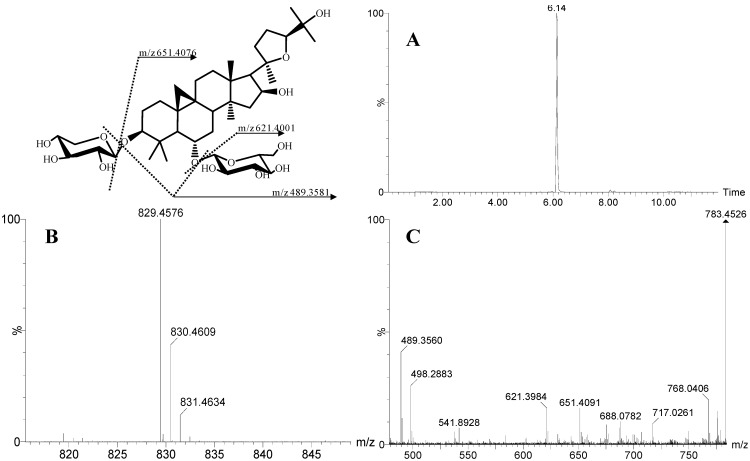
The XICs (**A**), MS (**B**) and MS/MS (**C**) spectra of astragaloside IV.

ESI mass spectra of positive and negative patterns were obtained. We elicited more information on the product ions of the metabolites of astragaloside IV in negative ion mode than in positive ion mode. MS/MS fragmentation of the molecular ions of astragaloside IV was obtained with a standard of astragaloside IV. The peak that eluted at 6.14 min appeared to have the same molecular ion, MS-fragmentation, and chromatographic behavior as astragaloside IV (Figure 1). Full-scan MS analyses of astragaloside IV showed strong [M+HCOOH‒H]^−^ at *m/z* 829.4567 and two isotope peaks (*m/z* 830.4609 and *m/z* 831.4634). The MS/MS of astragaloside IV showed a peak at *m/z* 651.4076, which demonstrated a loss of xylose from the parent ion. Another ion at *m/z* 621.4001 was caused by the loss of a glucose molecule. The product ion at *m/z* 489.3581 was 132 Da less than *m/z* 621.4001, suggesting the loss of xylose. Accordingly, ions at *m/z* 651.4076, *m/z* 621.4001 and *m/z* 489.3581 were the characteristic product ions of astragaloside IV. Also, 162 Da and 132 Da were the characteristic neutral losses of astragaloside IV. These characteristic product ions and neutral losses were essential for identification of the metabolites of astragaloside IV.

### 2.2. Metabolite Profiling

**Figure 2:**
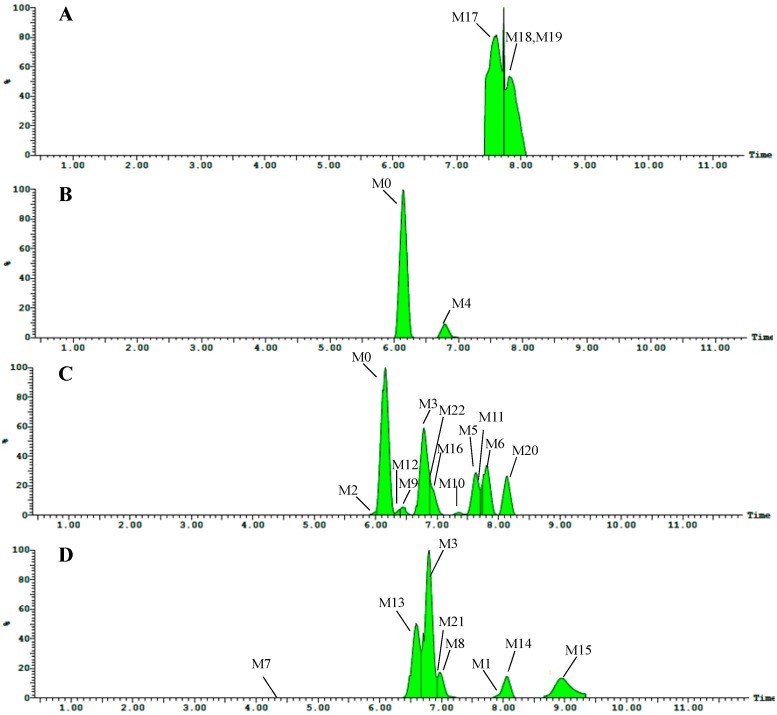
Representative TIC chromatograms of rat samples after intragastric administration of astragaloside IV at 80 mg/kg. (**A**) plasma; (**B**) bile; (**C**) urine; (**D**) feces.

**Figure 3:**
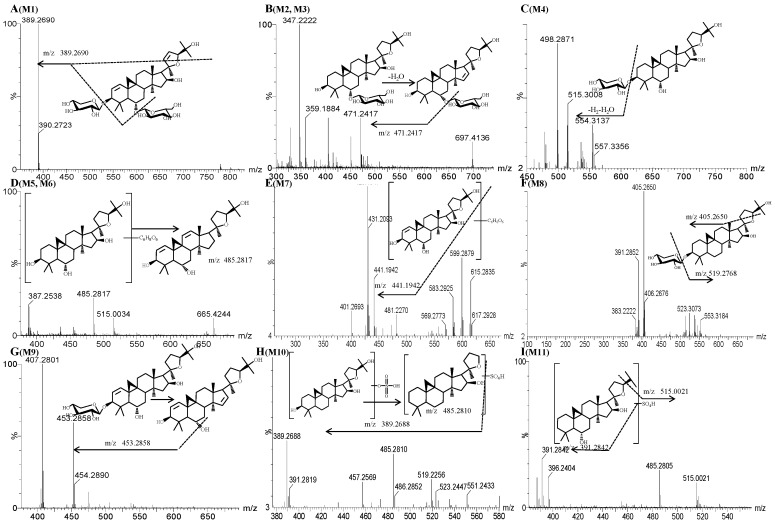

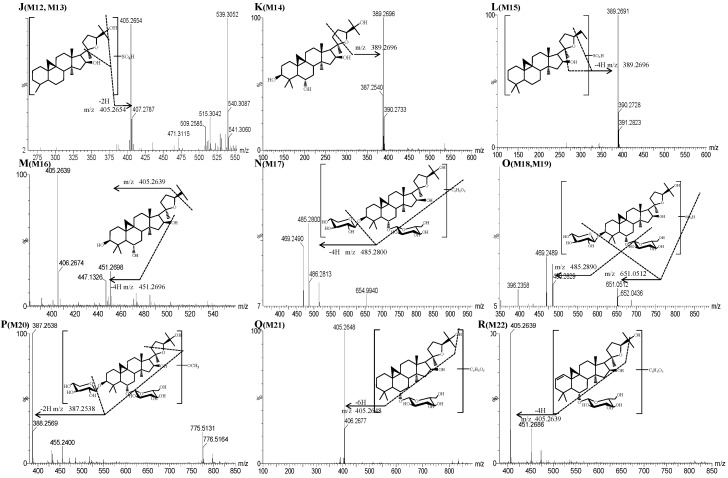
Mass spectra (MS/MS) of the metabolites of astragaloside IV.

**Figure 4:**
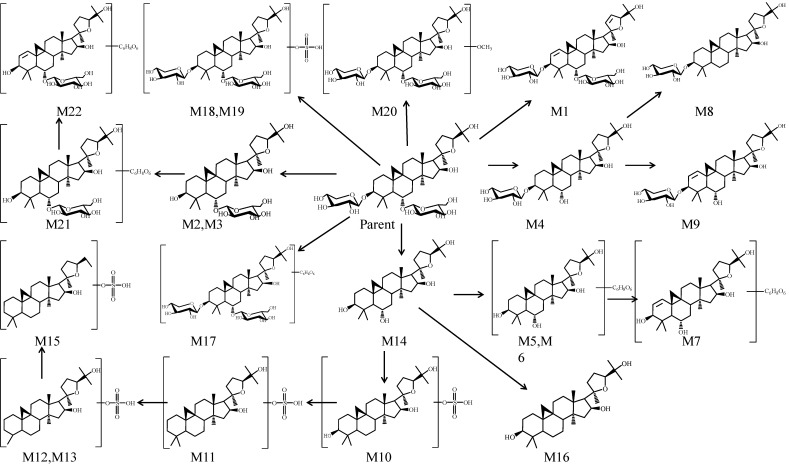
Proposed major metabolic pathway of astragaloside IV.

Twenty-two metabolites of astragaloside IV were detected by UPLC-Q-TOF-MS. Sixteen metabolites (M1 and M8–M22) were new. Information regarding these metabolites (retention times, elemental compositions proposed, characteristic fragment ions) is shown in Table 1. The TIC chromatograms of plasma, bile, urine and feces samples in the negative ion mode are shown in Figure 2, respectively. TOF/MS spectra of these metabolites are shown in Figure 3. Structures of the metabolites were obtained by comparing the characteristics of mass spectral fragmentation of metabolites with the literature or corresponding standards. Hydrolysis, glucuronidation, sulfation and dehydrogenation were the main metabolic pathways of astragaloside IV in rats (Figure 4).

### 2.3. Identification of Metabolites

#### 2.3.1. Metabolite M1

Metabolite M1 had a retention time of 7.98 min. The [M−H]^−^ molecular ion of metabolite M1 had a weight of 779.4278 Da, which suggested a molecular formula of C_41_H_64_O_14_. The molecular weight of M1 was 50 Da less than that of the parent drug, and showed that M1 was formed by loss of 46 Da (formic acid) along with 4 Da (di-dehydrogenation) of the parent drug. The fragment ion of M1 at *m/z* 389.2690 was formed by the loss of glucose and xylose as well as breaking of the furan ring from the precursor ion (*m/z* 779.4278). Hence, M1 was identified as a di-dehydrogenation metabolite of astragaloside IV. The mass spectrum of the di-dehydrogenation metabolite is shown in Figure 3A.

#### 2.3.2. Metabolites M2 and M3

Metabolites M2 and M3 had retention times of 6.00 min and 6.77 min, respectively. The [M+HCOOH‒H]^−^ molecular ion of metabolites had a weight of 697.4193 Da, which heralded a molecular formula of C_37_H_62_O_12_. Compared with the literature, the molecular weight of metabolites was 132 Da less than that of the parent drug, demonstrating that these metabolites were dexylcosylation metabolites of astragaloside IV. The fragment ion at *m/z* 471.2417 was formed by the loss of 46 Da (formic acid) along with 162 Da (hexose) and 18 Da water from the precursor ion (*m/z* 697.4193), which suggested the loss of glucose and dehydration. Hence, M2 and M3 were identified as isomers of dexylcosylation products of astragaloside IV. However, the detailed mechanisms for the formation of isomeric remain unknown. The mass spectra of M2 and M3 are shown in Figure 3B.

**Table 1:** Identification of astragaloside IV in rat samples using UPLC-Q-TOF-MS.

No.	Ion mode	Formula	t_R_(min)	Calculated(*m/z*)	Measured(*m/z*)	ppmerror	Fragments(*m/z*)	Metabolic Description	Metabolites Found			
									Plasma	Bile	Urine	Feces
M0	[M+HCOOH−H]^−^	C_42_H_70_O_16_	6.14	829.4586	829.4562	−2.9	621, 489	Parent	No	Yes	Yes	No
M1	[M−H]^−^	C_41_H_64_O_14_	7.98	779.4218	779.4278	7.7	389.2690	dehydrogenation	No	No	No	Yes
M2	[M+HCOOH−H]^−^	C_37_H_62_O_12_	6.00	697.4163	697.4193	4.3	471.2417, 359.1884, 347.2222	Deglycosylation	No	No	Yes	No
M3	[M+HCOOH−H]^−^	C_37_H_62_O_12_	6.77	697.4163	697.4145	−2.6	471.2417, 359.1884, 347.2222	Deglycosylation	No	No	Yes	Yes
M4	[M+HCOOH−H]^−^	C_36_H_60_O_11_	6.78	667.4058	667.4044	−2	515.3008	Deglycosylation	No	Yes	No	No
M5	[M−H]^−^	C_37_H_62_O_10_	7.62	665.4265	665.4269	0.6	485.2821	Glucuronide + deglycosylation	No	No	Yes	No
M6	[M−H]^−^	C_37_H_62_O_10_	7.8	665.4265	665.4237	−4.2	485.2817	Glucuronide + deglycosylation	No	No	Yes	No
M7	[M−H]^−^	C_36_H_56_O_11_	4.32	663.3745	663.3753	1.3	615.2835, 599.2897, 583.2925, 429.1947	Glucuronide + deglycosylation	No	No	No	Yes
M8	[M+HCOOH−H]^−^	C_36_H_60_O_10_	6.96	651.4108	651.4109	0.1	553.3184, 523.3073, 405.2650, 391.2852	Deglycosylation + dehydroxylation	No	No	No	Yes
M9	[M−H]^−^	C_36_H_60_O_8_	6.43	619.421	619.4197	−2.1	453.2858, 407.2801	Deglycosylation + dehydrogenation	No	No	Yes	No
M10	[M−H]^−^	C_30_H_50_O_8_S	7.34	569.3148	569.3162	2.4	551.2433, 519.2256,485.2810, 457.2569	Deglycosylation + sulfation	No	No	Yes	No
M11	[M−H]^−^	C_30_H_50_O_7_S	7.74	553.3199	553.3212	2.3	515.0021, 485.2805	Deglycosylation + sulfation	No	No	Yes	No
M12	[M−H]^−^	C_29_H_48_O_7_S	6.4	539.3043	539.3059	3	475.2653, 453.2846, 407.2786, 405.2641	Deglycosylation + sulfation+ demethylation	No	No	Yes	No
M13	[M−H]^−^	C_29_H_48_O_7_S	6.58	539.3043	539.3036	−1.2	515.3042, 509.2585, 471.3115, 405.2654	Deglycosylation + sulfation + demethylation	No	No	No	Yes
M14	[M+HCOOH−H]^−^	C_31_H_52_O_7_	8.05	535.3635	535.364	0.9	389.2696	Deglycosylation	No	No	No	Yes
M15	[M−H]^−^	C_29_H_48_O_6_S	8.93	523.3094	523.3094	0.1	389.2691	Deglycosylation + sulfation + demethylation + deoxygenation	No	No	No	Yes
M16	[M−H]^−^	C_29_H_46_O_5_	6.89	473.3267	473.3263	−0.9	451.2698, 447.1326, 405.2639	Deglycosylation + deoxygenation	No	No	Yes	No
M17	[M−H]^−^	C_46_H_76_O_20_	7.91	959.4852	959.487	1.9	485.2800, 469.2490	Glucuronidation	Yes	No	No	No
M18	[M−H]^−^	C_42_H_72_O_17_S	7.62	879.4412	879.4387	−2.9	651.0512, 469.2489	Sulfateconjugation	Yes	No	No	No
M19	[M−H]^−^	C_42_H_72_O_17_S	7.81	879.4412	879.4373	−3.9	651.0512, 469.2489	Sulfateconjugation	Yes	No	No	No
M20	[M−H]^−^	C_42_H_70_O_15_	8.14	813.4637	813.4667	3.7	775.5131, 455.2400, 387.2538	Methoxylation	No	No	Yes	No
M21	[M−H]^−^	C_41_H_64_O_16_	6.92	811.4116	811.4180	7.8	405.2648	Glucuronide + deglycosylation	No	No	No	Yes
M22	[M−H]^−^	C_42_H_66_O_15_	6.86	809.4324	809.4262	−7.6	451.2686, 405.2628	Glucuronide + deglycosylation + dehydrogenation	No	No	Yes	No

#### 2.3.3. Metabolite M4

The molecular ion detected at *m/z* 667.4044 Da (M4, [M+HCOOH−H]^−^) was eluted at a retention time of 6.78 min. Metabolite M4 had a molecular formula of C_36_H_60_O_11_, which was 162 Da less than that of the parent drug. Comparison with the literature revealed M4 to be a hydrolyzed metabolite with deglycosylation of the parent drug. The fragment ion of M4 at *m/z* 515.3008 was formed by loss of 152 Da from the precursor ion (*m/z* 667.4044). This finding suggested the loss of 132 Da (pentose) and 18 Da water and 2 Da hydration and dehydrogenation, which further confirmed the conclusion made above. Hence, M4 was identified to be a deglycosylation metabolite of astragaloside IV. The mass spectrum is shown in Figure 3C.

#### 2.3.4. Metabolites M5 and M6

Metabolites M5 and M6 had retention times of 7.62 min and 7.80 min, respectively. The [M−H]^−^ molecular ions of metabolite M5 and M6 had a weight of 665.4269 Da, which heralded a molecular formula of C_37_H_62_O_8_. The molecular weights of M5 and M6 were 164 Da less than that of the parent drug. Compared with literature, M5 and M6 were glucuronidation and deglycosylation metabolites of the parent drug [15]. The fragment ion at *m/z* 485.2821 was formed by the loss of glucuronidation and dehydrogen. Hence, M5 and M6 were identified as glucuronidation dehydroxylation and deglycosylation metabolites of astragaloside IV. Mass spectra of the glucuronidation dehydroxylation and deglycosylation metabolites are shown in Figure 3D.

#### 2.3.5. Metabolite M7

The molecular ion detected at *m/z* 663.3753Da (M3, [M‒H]^−^) was eluted at a retention time of 4.32 min. The metabolite M7 had a molecular formula of C_36_H_56_O_11_, which had a weight that was 2 Da less than that for M5 and M6. Comparison with the literature revealed M7 to be the glucuronidation, deglycosylation and dehydrogenation metabolite of astragaloside IV. The fragment ion of M7 at *m/z* 615.2835 was formed by loss of 46 Da (formic acid) along with 2 Da (hydrogen) from the precursor ion (*m/z* 663.3753). The ions at *m/z* 599.2879 and *m/z* 583.2925 were caused by deoxygenation. The ion at *m/z* 441.1942 was formed by the loss of glucuronate, which further confirmed the conclusion made above. Hence, M7 was identified as the glucuronidation, dehydroxylation, deglycosylation and dehydrogenation metabolite of astragaloside IV. The mass spectrum is shown in Figure 3E.

#### 2.3.6. Metabolite M8

Metabolite M8 had a retention time of 6.96 min. The [M+HCOOH−H]^−^ molecular ion of metabolite M8 had a weight of 651.4109 Da, which heralded a molecular formula of C_36_H_60_O_10_. The molecular weight of M8 was 178 Da less than that of the parent drug. The MS/MS fragment ion of M8 at *m/z* 519.2768 was formed by the loss of C_5_H_8_O_4_ (132 Da) from the precursor ion (*m/z* 651.4109). The fragment at *m/z* 519.2768 indicated the loss of xylose and a methyl group. Hence, M8 was identified as a deglycosylation and dehydroxylation metabolite of astragaloside IV. The mass spectrum of M8 is shown in Figure 3F.

#### 2.3.7. Metabolite M9

The molecular ion detected at *m/z* 619.4197 (M9, [M−H]^−^) was eluted at a retention time of 6.43 min. Metabolite M9 had a molecular formula of C_36_H_60_O_8_, which carried a weight 210 Da less than that of the parent drug. M9 was confirmed to be loss of 46 Da (formic acid) along with the deglycosylation and dehydrogenation metabolite of the parent drug. The fragment ion of M9 at *m/z* 453.2858 was formed by the loss of xylose, oxygen and dehydratation from the precursor ion (*m/z* 619.4197). The ion produced at *m/z* 407.2801 was formed by the loss of hydroxy and methyl groups. Ion information further confirmed our conclusion. Hence, M9 was identified as a deglycosylation and dehydrogenation metabolite of astragaloside IV. The mass spectrum is shown in Figure 3G.

#### 2.3.8. Metabolite M10

The molecular ion detected at *m/z* 569.3162 Da (M10, [M−H]^−^) was eluted at a retention time of 7.34 min. Metabolite M10 had a molecular formula of C_30_H_50_O_8_S, which was a weight of 260 Da less than that of the parent drug. The fragment ion of M10 at *m/z* 551.2433 was formed by the loss of hydration from the precursor ion (*m/z* 569.3162). The ion at *m/z* 519.2256 was caused by the loss of two hydroxyl groups from the ion (*m/z* 551.2433). The fragment ion of *m/z* 485.2810 carried a weight that was 34 Da less than the ion at *m/z* 519.2256, which was caused by the loss of a hydroxy group and hydration. The fragment ion of M10 at *m/z* 389.2687 indicated the loss of a sulfate group from the precursor ion (*m/z* 485.2810). Hence, M10 was identified as a deglycosylation and sulfation metabolite of astragaloside IV. The mass spectrum is shown in Figure 3H.

#### 2.3.9. Metabolite M11

The molecular ion detected at *m/z* 553.3212 Da (M11, [M−H]^−^) was eluted at a retention time of 7.74 min. Metabolite M11 had a molecular formula of C_30_H_50_O_7_S, which carried a weight that was 16 Da less than that of M10. The fragment ion of M11 at *m/z* 515.0021 was formed by the loss of an isopropyl group (C_3_H_6_; 42 Da) from the precursor ion (*m/z* 553.3212). The ion at *m/z* 485.2805 was formed by the loss of methyl and hydroxy groups from ion *m/z* 515.0021. The fragment ion of M11 at *m/z* 391.2842 was the isoform of the ion that lost a sulfate group from the precursor ion (*m/z* 485.2810). Hence, M11 was identified as a deglycosylation and sulfation metabolite of astragaloside IV. The mass spectrum is shown in Figure 3I.

#### 2.3.10. Metabolites M12 and M13

The molecular ion detected at *m/z* 539.3059 Da (M12, [M−H]^−^) was eluted at a retention time of 6.40 min. Metabolite M12 had a molecular formula of C_29_H_48_O_7_S, which is a weight of 14 Da less than that of M11, suggesting that it was probably produced by neutral loss of a methyl group. The fragment ion at *m/z* 405.2654 was caused by loss of sulfate and methyl groups as well as a hydroxyl group associated with dehydrogenation. Therefore, M12 was identified as a hydrolyzed metabolite that underwent deglycosylation, sulfation and demethylation of the parent drug. The mass spectrum is shown in Figure 3J.

#### 2.3.11. Metabolite M14

Metabolite M14 had a retention time of 8.05 min. The [M+HCOOH−H]^−^ molecular ions of metabolite M14 carried a weight of 535.3640 Da, which heralded a molecular formula of C_31_H_52_O_7_. The molecular weight of M14 was 294 Da less than that of the parent drug, suggesting that M14 was a metabolite formed by loss of 46 Da (formic acid) along with di-deglycosilation and deglycosylation of the parent drug. The fragment ion of M14 at *m/z* 389.2696 was formed by the loss of hydroxyl and methyl groups from the precursor ion (*m/z* 535.3640). Hence, M14 was identified as the hydrolyzed metabolite of astragaloside IV. The mass spectrum is shown in Figure 3K.

#### 2.3.12. Metabolite M15

Metabolite M15 had a retention time of 8.93 min. The molecular weight of metabolite M15 was 523.3094 Da, which heralded a molecular formula of C_29_H_48_O_6_S. The molecular weight of M15 was 16 Da less than that of M12 and M13, suggesting that M15 was a dehydroxylation metabolite of M12 or M13. The fragment ion of M15 at *m/z* 389.2691 was caused by the loss of sulfate and methyl groups and a hydroxyl group associated with dehydrogenation from the precursor ion (*m/z* 523.3094), which further confirmed the above conclusion. Hence, M15 was identified as a deglycosylation, sulfation, demethylation and hydroxylation metabolite of astragaloside IV. The mass spectrum of M15 is shown in Figure 3L.

#### 2.3.13. Metabolite M16

The molecular ion detected at *m/z* 473.3263 Da (M16, [M−H]^−^) was eluted at a retention time of 6.89 min. Metabolite M16 had a molecular formula of C_29_H_46_O_5_, the weight of which was 356 Da less than that of the parent drug. The fragment ion of M16 at *m/z* 451.2698 was formed by dehydration and dehydrogenation. The fragment ion at *m/z* 405.2636 was formed by the loss of an isopropyl group. Hence, M16 was identified as a deglycosylation and dehydroxylation metabolite of astragaloside IV. The mass spectrum is shown in Figure 3M.

#### 2.3.14. Metabolite M17

Metabolite M17 had a retention time of 7.91 min. The molecular ions of metabolite M17 had a weight of 959.4852 Da, which heralded a molecular formula of C_46_H_76_O_20_. The molecular weight of M17 was 130 Da greater than that of the parent drug, suggesting that M17 was a metabolite formed by loss of 46 Da (formic acid) along with glucuronation of the parent. The fragment ion at *m/z* 485.2800 was formed by the loss of glucuronic acid, glucose, hydrogen and xylose from the precursor ion (*m/z* 959.487). The fragment ion at *m/z* 469.2490 was formed by dehydroxygenation of the ion at *m/z* 485.2800. Hence, M17 was identified as a glucuronation and demethylation metabolite of astragaloside IV. The mass spectrum is shown in Figure 3N.

#### 2.3.15. Metabolites M18 and M19

Metabolites M18 and M19 had retention times of 7.81 and 7.62 min, respectively. The molecular weight of metabolite M18 and M19 was 879.4373 Da, which heralded a molecular formula of C_41_H_68_O_17_S. The molecular weight of M18 and M19 was 50 Da larger than that of the parent drug, suggesting that M18 and M19 were isomers of the parent drug formed by loss of 46 Da (formic acid) along with sulfate conjunction metabolites. The fragment ion at *m/z* 651.0512 was formed by the loss of SO_4_ (96 Da) and xylose (132 Da) from the precursor ion (*m/z* 879). The fragment ion at *m/z* 651.0512 lost a glucose molecule (162 Da) and four hydrogen atoms (4 Da) to form the ion at *m/z* 485.2690. Hence, M18 and M19 were identified as sulfation metabolites of astragaloside IV. The mass spectra of the metabolites are shown in Figure 3O.

#### 2.3.16. Metabolite M20

Metabolite M20 had a retention time of 8.14 min. The molecular weight of metabolite M20 was 813.4667 Da, which heralded a molecular formula of C_42_H_70_O_15_. The molecular weight of M20 was 16 Da less than that of the parent drug, suggesting that M20 was a metabolite formed by loss of 46 Da (formic acid) along with methoxylated the metabolite of the parent drug. The fragment ion of M20 at *m/z* 775.5131 was formed by the loss of hydrogen atoms and hydroxyl groups from the precursor ion (*m/z* 813.4667). The fragment ion at *m/z* 387.2538 was formed by the loss of glucose, xylose, methyl and hydroxyl, which further confirmed the above conclusion. Hence, M20 was identified as a methoxylated metabolite of astragaloside IV. The mass spectrum is shown in Figure 3P.

#### 2.3.17. Metabolite M21

The molecular ion detected at *m/z* 811.4180 Da (M21, [M−H]^−^) was eluted at a retention time of 6.92 min. Metabolite M21 had a molecular formula of C_42_H_68_O_15_, which carried a molecular weight that was 18 Da less than that of the parent drug. The fragment ion of M21 at *m/z* 405.2648 was formed by the loss of glucose, glycuronic acid and isopropanol, with cleavage of the furan ring at sites 22 and 23 (406 Da) from the precursor ion (*m/z* 811.4180), which further confirmed the above conclusion. Hence, M21 was identified as a dexylcosylation and glucuronation metabolite of astragaloside IV. The mass spectrum is shown in Figure 3Q.

#### 2.3.18. Metabolite M22

The molecular ion detected at *m/z* 809.4262 Da (M22, [M+HCOOH−H]^−^) was eluted at a retention time of 6.86 min. Metabolite M22 had a molecular formula of C_42_H_66_O_15_, which carried a molecular weight that was 20 Da less than that of M21. The fragment ion of M22 at *m/z* 405 was formed by the loss of formic acid, glucose, glycuronic acid and isopropanol, along with cleavage of the furan ring at sites 22 and 23 (404 Da) from the precursor ion (*m/z* 809.4262), which further confirmed the above conclusion. Hence, M22 was identified as a dehydrogenation metabolite of M21. The mass spectrum is shown in Figure 3R.

In general, there were nine phase I metabolites of astragaloside IV in rats. The main processes were deglycosylation, dehydrogenation and hydroxylation. The phase II metabolites of astragaloside IV in rats included one methylation product (M20), six glucuronides (M5, M6, M7, M17, M21, M22) and seven sulphation ones (M10–M13, M15,M18, M19). The possible metabolic pathways of astragaloside IV are summarized in Figure 4.

### 2.4. Discussion

An UPLC-Q-TOF-MS/MS-based approach was applied to the metabolite profiling of astragaloside IV in plasma, bile, urine and feces samples from rats. A total of 22 metabolites was detected and tentatively identified, and 15 of them were reported for the first time. The major metabolic pathways of astragaloside IV in rats were deglycosylation, sulphation and glucuronidation. Physicochemical characteristics such as high molecular weight, high hydrogen-bonding capacity, high molecular flexibility, and poor membrane permeability may contribute to the poor bioavailability of saponins [17,18]. Studies have shown the saponins have poor absorption through the gut and limited metabolism by intestinal microflora compared with other compounds isolated from TCMs [19]. Additionally, astragaloside IV is metabolized into the aglycon or deglycosylated in the gastrointestinal tract and absorbed only partially. Several isomers cannot be discriminated from each other, so more advanced technology, such as LC-NMR-MS, is needed for confirmation.

## 3. Experimental Section

The study protocol (including all animal experiments) was approved by the Ethical Committee of Nanjing University of Chinese Medicine (Nanjing, China).

### 3.1. Chemicals and Reagents

Astragaloside IV (purity >98% as determined by high-performance liquid chromatography (HPLC)) was purchased from the National Institute for the Control of Pharmaceutical and Biological Products (Beijing, China). Formic acid, acetonitrile and methanol (Merck, Darmstadt, Germany) were of HPLC grade. Water was produced by a Milli-Q Ultrapure Water system (Millipore, Billerica, MA, USA). Sodium carboxyl methyl cellulose (CMC-Na) was supplied by Sinopharm Chemical Reagents (Beijing, China).

### 3.2. Animal Protocol

Male Sprague-Dawley (SD) rats (250 ± 20 g) were acquired from SLAC (Shanghai, China). Rats were kept in controlled-environment room (25 ± 2°C; humidity, 50% ± 10%; 12-h dark-light cycle) for ≥1 week for adaptation. Animals were fasted overnight before experimentation.

#### 3.2.1 Plasma Sampling

Astragaloside IV suspended in 0.5% CMC-Na was administered to three rats (80 mg/kg body weight, p.o.) on three consecutive occasions over 24 h. Before administration, blank blood samples were collected. The sampling time point of astragaloside IV in rat plasma was chosen as reported [15]. Briefly, blood samples were collected by sampling of the orbital sinus 180 min after the previous administration. Blood samples (0.5 mL) were collected into polyethylene (PE) with heparin-pretreated tubes. All blood samples were centrifuged for 10 min at 3000 g and 4 °C. Separated plasma samples were stored at –70 °C until additional extraction and analyses.

#### 3.2.2. Bile Sampling

Astragaloside IV was administered *via* the oral route to three rats at a single dose of ≈80 mg/kg. Animals were then anesthetized by 1% pentobarbital sodium (0.15 mL/100 g body weight; i.p.). An abdominal incision was made and the bile duct cannulated with PE-10 tubing (BD, Sparks, MD, USA) for collection of bile samples. A heating lamp was used for maintaining body temperature during experimental procedures to prevent hypothermic alterations. Drug-containing bile samples were collected for 24 h and stored at −70 °C until additional extraction and analyses. Meanwhile, blank bile samples were collected from an additional two rats.

#### 3.2.3. Sampling of Urine and Feces

Rats were placed in a metabolic cage for 24 h. Before experimentation, rats had been in metabolic cages for acclimatization for 2 days and given standard chow and water. Blank urine and feces samples were collected. Urine and feces samples were collected for 24 h. Rats were weighed and given astragaloside IV suspended in 0.5% CMC-Na (80 mg/kg body weight). Samples of urine and feces from three rats were collected for 48 h. All urine and feces samples were stored at −70 °C until additional extraction and analyses.

### 3.3. Sample Preparation

#### 3.3.1. Plasma

Plasma samples from individual animals were combined. After addition of 600 μL of methanol, plasma samples (200 μL) were vortex-mixed to precipitate plasma proteins, and then centrifuged at 3000 g for 10 min at 4 °C. Supernatants were loaded onto preconditioned C_18_ SPE columns. After washing with 1 mL of water, analytes were eluted with 1 mL methanol. Eluents were centrifuged at 28,000 g for 10 min at 4 °C. An aliquot (2 μL) of supernatants was then injected into the UPLCQ-TOFMS/MS system.

#### 3.3.2. Bile

Bile samples from individual animals were combined. Bile samples (500 μL) were loaded onto preconditioned C_18_ SPE columns directly. After washing with 1 mL of water, analytes were eluted with 1 mL methanol. Eluents were centrifuged at 28,000 g for 10 min at 4 °C. Supernatants (2 μL) were injected into the UPLCQ-TOFMS/MS system.

#### 3.3.3. Urine

Urine samples from individual animals were combined. The C_18_ SPE column was preconditioned with 2 mL methanol and 2 mL water. Urine samples (500 μL) were added onto the SPE columns. After washing with 1 mL water, analytes were eluted with 1 mL methanol. Eluents were centrifuged at 28,000 g for 10 min at 4 °C. Supernatants (2 μL) were injected into the UPLC-Q-TOF-MS/MS system.

#### 3.3.4. Feces

Feces (0.5 g) from each blank feces sample and drug-containing sample were selected separately. Methanol (5 mL) was added and the suspension vortex-mixed for 5 min, followed by ultrasonic extraction for 30 min using a KQ5200 Ultrasonic Cleaner (Kunshan Ultrasonic Instruments, Kunshan, Jiangsu, China). The mixture was centrifuged at 3000 g for 10 min at 4 °C to precipitate insoluble content. The supernatant was dried with nitrogen gas at 37 °C. Subsequently, 1 mL methanol was added to each sample followed by ultrasonic extraction for 20 min and extracts centrifuged at 28,000 g for 10 min at 4 °C. Supernatants were passed through 0.45-μm membranes (Millipore, Bedford, MA, USA) and injected into the UPLC-Q-TOF-MS/MS system.

### 3.4. Instrumentation and Conditions

UPLC separation was performed on an ACQUITY system (Waters, Milford, MA, USA) equipped with a binary solvent delivery system, auto-sampler and an ESI interface. Analytes were eluted on a ACQUITY BEH column (2.1 mm × 100 mm; 1.7 μm; Waters). The column temperature and auto-sampler temperature were maintained at 30 °C and 4 °C, respectively.

Analyses were undertaken on the ACQUITY system. The flow rate of the mobile phase was 0.3 mL/min and the volume injected was 2 μL. The mobile phase consisted of acetonitrile (A) and a 0.1% formic acid-water solution (B). Eluent gradients were: 5% A for 1 min; 5%–20% A from 1 min to 2 min; 20%–50% A from 2 min to 6 min; 50%–70% A from 6 min to 10 min; 70%–95% A from 10 min to 11 min; 95% A from 11 min to 11.5 min; 95% A from 11.5 min to 12 min.

A Xevo G2 Q-TOF Mass Spectrometer (Waters) was used in negative ESI mode for data acquisition using UPLC/MS^E^. The conditions of ESI-Q-TOF-MS/MS analyses were: capillary voltage, –2.5 kV; sample cone, 30 V; extraction cone, 4.0 V; source temperature, 120 °C; desolvation temperature 450 °C; flow rate of cone gas, 50 L/h; flow rate of desolvation gas (N_2_), 800 L/h.

The mass instrument was calibrated using sodium formate. Mass accuracy and reproducibility were maintained using a Lock Spray^TM^ interface. The [M−H]^−^ of leucine-enkephalin infused at 20 μL/min was used as a reference lock mass (*m/z* 554.2615 Da) at 200 pg/μL. Centroided data were acquired for each sample from 100 Da to 1500 Da. Dynamic range enhancement was applied throughout the MS experiment to ensure accurate mass measurements over a wide dynamic range. Accurate mass and elemental composition for precursor ions and fragment ions were analyzed using Mass Lynx v4.1.

### 3.5. Data Analyses

Data analyses were carried out with MetaboLynx. Analytical results of drug-contained samples and control were compared using MetaboLynx with a list of potential metabolites. Metabolites were detected and identified automatically.

## 4. Conclusions

The metabolite of astragaloside IV in rat plasma, bile, urine and feces samples were studied by UPLC-Q-TOF-MS/MS method. After adminitstration, astragaloside IV suffered with phases I and II metabolic reactions and generated 22metabolites were detected, 15 of them were reported for the first time. This study has improved our understanding of the metabolic profiling of astragaloside IV *in vivo*, and information gained from the present study is relevant to the pharmacological activity of astragaloside IV.
